# Evaluating the Relationship Between Electrical Dynamic Range and Speech Perception Outcomes in Experienced Post-Lingually Deaf Adult Cochlear Implant Users: A Bicentric Study

**DOI:** 10.3390/audiolres16020031

**Published:** 2026-02-25

**Authors:** Pietro Salvago, Davide Vaccaro, Fulvio Plescia, Francesca Di Marco, Sabrina Loteta, Daniele Portelli, Giuseppe Alberti, Francesco Dispenza, Francesco Freni, Pasquale Riccardi, Francesco Martines

**Affiliations:** 1 Dipartimento di Biomedicina, Neuroscienze e Diagnostica Avanzata (BiND), Sezione di Audiologia, Università degli Studi di Palermo, Via del Vespro 129, 90127 Palermo, Italy; 2 Dipartimento di Promozione della Salute, Materno-Infantile, di Medicina Interna e Specialistica di Eccellenza “G. D’Alessandro”, Università degli Studi di Palermo, Via del Vespro 133, 90127 Palermo, Italy; 3 Advanced Bionics Italia, Via Privata Raimondo Montecuccoli, 30, 20147 Milan, Italy; 4 Dipartimento di Patologia Umana dell’Adulto e dell’Età Evolutiva “Gaetano Barresi”, Università degli Studi di Messina, Via Consolare Valeria 1, 98125 Messina, Italy; lotetas@unime.it (S.L.); galberti@unime.it (G.A.);; 5 Dipartimento di Biomedicina, Neuroscienze e Diagnostica Avanzata (BiND), Sezione di Otorinolaringoiatria, Università degli Studi di Palermo, Via del Vespro 129, 90127 Palermo, Italy; francesco.dispenza@unipa.it

**Keywords:** cochlear implant, dynamic range, speech perception, speech curve, EDR

## Abstract

Objectives: To analyze speech perception outcomes of a cohort of experienced adult cochlear implant (CI) users to explore whether there is a correlation with electrical dynamic range (EDR) parameters, and to describe speech intelligibility curve morphology according to the degree of CI performance. Methods: A bicentric retrospective observational study. Data were extracted from a cochlear implantation database from a total of 36 CI users implanted with Advanced Bionics devices. Results: Mean age at implantation was 56.61 years. In the majority of cases, hearing loss onset was more than 15 years before implantation (80.55%), and only 11.11% of cases preserved residual hearing. This resulted in a significant relationship between speech therapy and better speech recognition (*p* = 0.044). At the same time, no correlation was found between age, duration of deafness before implantation, and maximum speech perception achieved (*p* > 0.05). Mean speech audiometry curves displayed a roll-over phenomenon in poor performers and a plateau effect in average performers. In contrast, the mean curve of high performers exhibited a steeper morphology (*p* < 0.0001). Speech recognition threshold (SRT) and word recognition score (WRS) were predictors of speech audiogram curves (*p* = 0.006). No direct correlation was found between the mean T-level, M-level, dynamic range, and maximum recognition score, even after clustering electrodes by position along the cochlea (*p* > 0.05). Conclusions: EDR parameters did not emerge as independent predictors of speech recognition outcomes within this specific cohort. Speech therapy and rehabilitative efforts showed a significant relationship with improved performance, and speech audiogram curve morphology may offer a more specific clinical tool for assessing global CI performance. Further prospective studies with larger, more homogenous populations are required to validate these findings.

## 1. Introduction

Cochlear implantation has become the option of choice for managing severe to profound hearing loss when hearing aids are unable to help patients achieve good speech intelligibility [1,2]. Its application is progressively expanding, including for specific hearing loss conditions (e.g., single-sided deafness, asymmetric hearing loss, ski-slope hearing loss) that were previously not considered [3,4,5]. However, despite less stringent candidacy criteria, unexplained high variability in speech perception outcomes persists [6,7]. Many researchers have sought to address this issue, identifying different patient-related factors, intraoperative conditions, and post-surgical rehabilitation pitfalls, with heterogeneous results [8,9,10,11,12]. Additionally, post-lingually adult cochlear implant (CI) users present specific expectations and needs from prelingual deafened individuals because they often experience a mismatch between long-term stored phonological memories and the “new” incoming signal from CI [13]. Furthermore, they show various degrees of residual neuroplasticity that may be related to the duration of deafness and their age at the time of surgery [14].

Speech intelligibility has been assessed with different tests under quiet and noisy conditions, and it gives the audiologist an objective measure of a CI recipient’s performance, though it does not consider subjective and psychological factors that may be better assessed through benefit questionnaires. In fact, some authors demonstrated no significant or weak correlation between speech recognition percentages and the results of self-assessment questionnaires [15,16,17].

An interesting field of research concerns how the fitting of CIs affects users’ speech recognition abilities. From post-surgical activation of the speech processor to regular follow-up appointments, the audiologist is involved in a continuous process of adjusting the so-called “map,” a program composed of many variables that govern how sounds are translated into electrical pulses [18]. Fitting a CI is a process based on several variables, with different centers adopting their own timeline, measures, and targets, as evidence-based guidelines do not yet exist [19].

As in patients with sensorineural hearing loss (SNHL), where increased hearing thresholds and reduced dynamic range can often lead to impaired speech intelligibility, CI users may also experience negative effects on speech recognition due to variations in the map’s upper and lower stimulation levels. The dynamic range of CIs includes both electric dynamic range (EDR) and input dynamic range (*IDR*). The former is defined as the difference between the highest tolerable current level, without discomfort or pain (typically called a C-level, M-level, or MCL), and the perceptual threshold for sound (typically called a T-level or THR) [20]. Theoretically, a narrower EDR implies compressing a large acoustic dynamic range into a smaller electrical dynamic range. Excessive compression can distort the speech envelope or alter modulation depth, reducing temporal resolution and potentially impairing the user’s ability to perceive changes in loudness. Conversely, a broader EDR reduces the degree of such compression and may permit more proportional mapping between acoustic inputs and electrical outputs.

Previous research on the effects of stimulation levels and EDR on speech perception in adult CI recipients has revealed a negative influence on the part of restricted EDR, although significant effects have not been consistently reported [21,22,23].

On the other hand, *IDR* is the range of the incoming acoustic signal that is mapped onto the CI user’s EDR. Generally, a wide *IDR* will capture more of the incoming acoustic signal than a narrow *IDR*, allowing the CI user to hear soft, medium, and loud sounds [24]. A narrow *IDR* may restrict a CI user’s ability to hear soft speech and sounds because less of the incoming acoustic signal is being mapped onto the CI user’s EDR [25]. Because of the wide variation of loud speech, lowering *IDR* too much can hypothetically reduce speech comprehension, even in the absence of background noise.

The translation of acoustic sound pressure into electrical stimulation in CI systems requires a robust compression strategy to map the expansive acoustic dynamic range to the significantly narrower EDR of the human auditory nerve. The primary divergence between manufacturers lies in the mathematical nature of this mapping. For example, Cochlear employs a steep logarithmic compression characterized by the Q-value, while Advanced Bionics utilizes a relatively linear power-law function. In the Cochlear system, the Q-value defines the percentage of the *IDR* mapped to the upper portion of the electrical dynamic range. For a standard Q = 20, the mapping function is highly non-linear; the lower 90% of the acoustic input range is compressed into the upper approximately 40% of the electrical range. Visually, this creates a steep, convex curve in which the electrical output rises sharply from the T-level and plateaus at the C-level.

For Advanced Bionics, the *IDR* is programmable to 80 dB and assumes the microphone’s minimum signal is 25 dB. Therefore, by setting the *IDR* to its maximum, the microphone acquires sound signals at 25–105 dB SPL. The compression of the acoustic signal into an electrical signal involves two stages: for acoustic signals up to approximately 60 dB, the compression is linear; above 60 dB, it becomes logarithmic, with the slope depending on the selected *M* and *IDR* levels. Consequently, for Advanced Bionics, the electrical *T*-level corresponds to an acoustic signal intensity of 25 dB, while *M*-levels correspond to sound intensities equal to or greater than 25 + *IDR* dB.

The transduction function between the acoustic and electrical signals is as follows (provided by Advanced Bionics Italy, Milano, Italy):

$$I\;=\frac{\left(M-T\right)}{IDR}\times \left(L-55+IDR+GAIN\right)+T$$

where “*L*” represents the level defined by the envelope curve, “*GAIN*” represents the possible amplification of the channel, while the following relation gives the transformation in Clinical unit (CU):

$$\mathrm{CU}\;=\frac{Electric\;stimulation\left(\mu A\right)\times pulse\;wide(\mu s)}{1000}\times 0.0128447$$

Cochlear compression is similar to Advanced Bionics compression in that it provides linear compression up to a sound intensity level defined by the Q value, which represents the compression knee. Once the “Q” level is exceeded, the compression becomes logarithmic.

The transduction function between the acoustic and electrical signals is as follows (provided by Cochlear Italy, Bologna, Italy):

$$I\left(\mu A\right)=17.5\times 100^{\frac{CL}{255}}$$

while the transformation in current level (*CL*) is given by the following relation:

$$CL=\left(\log_{100}\frac{I}{17.5}\right)\times 255$$

The purpose of the present study was to retrospectively analyze speech perception outcomes in a cohort of experienced CI users to determine whether there is a correlation with EDR parameters. Specifically, we sought to evaluate the effect of EDR on speech intelligibility in quiet contexts, in terms of predicting the maximum speech recognition achieved and the curve of the speech diagram. To ensure better data homogeneity, all study subjects received identical CI devices (HiRes implants). A secondary aim was to describe the morphology of the speech intelligibility curve according to CI performance.

## 2. Materials and Methods

### 2.1. Participants

We conducted a bicentric, retrospective, observational study at the Policlinico “P. Giaccone” University Hospital of Palermo (Italy) and the Policlinico “G. Martino” University Hospital of Messina (Italy). Data were extracted from the cochlear implantation database of audiology diagnostic centers at the respective hospitals, where the CIs were activated, and patients were followed after surgery. We collected data from 36 CI users (22 women and 14 men) who were implanted between 2015 and 2023 using the Advanced Bionics HiRes 90K Advantage or HiRes Ultra with the HiFocus Mid-scala or SlimJ electrodes.

All subjects were post-lingually deafened adults 18 years or older at the time of implantation. To select a homogeneous cohort, subjects with cochlear ossification, inner ear malformations, single-sided deafness, severe neurologic and psychiatric disorders, and uncooperative patients were excluded from this study.

The participants suffered from bilateral SNHL and wore at least one CI on one side with a minimum of 2 years of follow-up. Specifically, 12 patients were unilateral CI users, 3 were bilaterally implanted, and 21 were bimodal users.

The Ethics Committee decided that no ethical approval statement was required for the study, considering its retrospective nature, lack of randomization, and the patient’s conscious choice of CI.

Details regarding patient demographics, etiology, duration of deafness in the implanted ear [26], post-activation speech therapy, and hours of CI use were collected. After careful anamnesis, patients underwent micro-otoscopy to rule out middle-ear pathologies and/or active infections. Pure-tone audiometry (PTA) was performed by a trained audiologist with a Piano clinical audiometer (Inventis, Padua, Italy) in a soundproof audiometric room. Air conduction was measured using an on-ear TDH-49 headphones set to 250–8000 Hz; bone conduction was measured using a calibrated bone transducer at 250–4000 Hz [27]. If no threshold could be determined on AC at given frequency, the threshold was set to 120 dB HL. To assess gain with a CI or hearing aid, we performed free-field measurement audiometry with warble stimuli via a FBT J5A loudspeaker (FBT Elettronica SpA, Recanati, Italy) placed 1 m from the participant at 0° azimuth.

Speech recognition was studied with a Piano clinical audiometer (Inventis, Padua, Italy) in a soundproof booth under quiet conditions, and tests were performed by experienced audiologists using standard clinical protocols. Speech recognition in quiet conditions was administered using an open-set, phonemically balanced word test. Ten meaningful disyllabic words were presented with the patient wearing the CI or the hearing aid using a FBT J5A loudspeaker (FBT Elettronica SpA, Recanati, Italy) placed 1 m from the participant at 0° azimuth; the test was performed at different intensities, from the softest audible speech signal up to 70 dB HL. Patients’ speech perception results were obtained at their most recent clinical evaluation, using each subject’s everyday program and most commonly used speech processor control settings. Aided listening conditions included only CI (for unilateral CI users), hearing aid alone and CI alone (for bimodal users), left ear only and right ear only (for bilateral CI recipients). If masking of the better hearing ear was required, the non-test ear was masked with speech noise using an insert earphone. Listeners were instructed to repeat each word. The percentage of correct answers (word recognition score, WRS) was calculated to determine the final score. The speech reception threshold (SRT) was defined as the minimum hearing level (dB HL) for speech at which an individual can recognize 50% of the speech material; according to the maximum percentage of speech discrimination achieved with CI, patients were classified in high (>70% of speech recognition), average (between 40 and 70%) and poor performers (<40%) [28].

### 2.2. Fitting Data

Participants wore a Naída Q70, Q90, or M90 sound processor depending on when the CI was implanted. Processor programming was performed by experienced CI audiologists using SoundWave™ (Version 3.2.12) and Target CI™ (Version 1.5) clinical software. HiRes Optima S was the speech coding strategy used.

The programming procedure for each participant followed the standard clinical protocol for all adult CI patients. This protocol included testing different stimulation rates and speech processing strategies, adjusting electrode threshold levels (T-levels) and highest comfort levels (M-levels), and modifying other parameters such as gain, the frequency assignment table, and the number of active electrodes.

Specifically, individual maps were created by defining the T-levels and M-levels for each active electrode. These parameters are fundamental to tailoring the electrical stimulation to the patient’s specific auditory range. The mapping procedure involved patients reporting sound loudness on a subjective scale ranging from barely audible to maximum comfort, with M-levels typically set at maximum comfort and T-levels at barely audible. While Advanced Bionics suggests setting T-levels to 10% of M-levels based on an assumed 20 dB electrical dynamic range, this study prioritized maximizing soft speech perception by measuring behavioral thresholds for each electrode to assign T-levels, acknowledging this is more time-consuming. The stimulus used in the clinical programming software consisted of “tone bursts”, specifically biphasic pulse trains with a duration dependent on the software programming and frequency dependent on the center frequency of the clinical programming channel. During this procedure, the electrical stimulus—measured in CUs—was initialized at zero for each electrode. The current was then progressively increased from this baseline. At the same time, the subject provided real-time feedback, identifying the perceived loudness as it transitioned from the point of initial detection to the level of maximum comfort. Furthermore, when the audiologist activates the implant, the summation effect of the global electrical stimulation is observed. For Advanced Bionics’ Optima Strategy, each channel has its own stimulation frequency to simulate the time coding of the cochlea, and thus the overall effect of the electrical stimulation is monitored on the patient’s loudness.

CI fitting also included setting the speech processing strategy, pulse width, gain, and the number of active electrodes for each individual to optimize speech intelligibility at conversational and soft levels; HiRes Optima S was the speech coding strategy used, and all subjects used the default 60 dB *IDR*. Patients in this study used between 14 and 16 active electrodes in their everyday program, depending on the electrodes removed during clinical programming. The EDR was calculated by measuring the difference between the T-level and the M-level for each electrode.

Patients showing a decline in speech recognition/sound quality, a change in impedances (typically <3.2 kOhms), elevated aided detection thresholds (typically high frequencies first), and/or alterations in electrically evoked compound action potentials underwent an integrity test to acquire electrical field imaging (EFI) changes and detect V1 failure [29]. This hard failure involved the initial version (V1) of HiRes Ultra and HiRes Ultra 3D cochlear implant (CI) devices due to a vulnerability of the implanted device to moisture; specifically, the fluid ingress may lead to partial short circuits to the implant’s reference electrodes, resulting in reduced amplitude stimuli and reduced sound quality [29]. Five subjects developed a V1 failure during follow-up; given the possible influence of V1 failure on speech perception outcomes, we included only data before V1 failure onset.

### 2.3. Statistical Analysis

Continuous variables were presented as mean ± standard deviation (SD), and categorical variables as numbers and percentages. Data normality was assessed using the Shapiro–Wilks normality test. Comparisons between categorical variables were performed using the Chi-square test and Fisher’s exact test. The Mann–Whitney U test was used to analyze continuous variables that were not normally distributed. Pearson’s correlation was used if normality and linearity were present; otherwise, Spearman’s rho was used. A simple logistic regression analysis was performed, treating the speech articulation curve as the dependent variable and SRT, WRS, T-level, M-level and dynamic range as independent variables. A two-tailed *p* < 0.05 was considered significant. STATISTICA software version. 8.0 (StatSoft. Inc., Tulsa, OK, USA) was utilized.

## 3. Results

**Figure 1:**
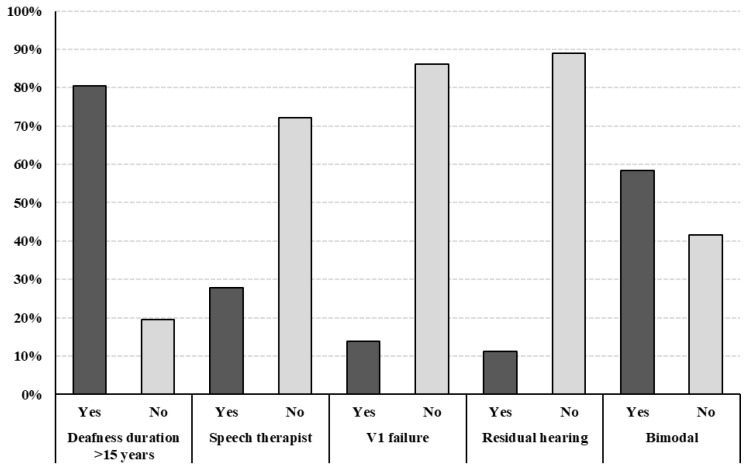
Pre- and post-implantation audiological features of the sample.

The mean age at time of implantation was 56.61 ± 13.37 years (age range 26–75), with 25% of patients being implanted at ≥70 years of age. The mean time of follow-up was 5.97 years, with a mean CI wear time of 11 h per day. Three individuals were bilaterally implanted, while 58.33% were right-side implanted and 33.33% were left-side CI users. In the majority of cases (63.88%), no certain hearing loss etiology was found. In contrast, in the remaining cases, we recognized Ménière’s disease in five, sudden SNHL in four, ear infections in two, otosclerosis in one, and genetic hearing loss in one patient, respectively. Hearing deterioration was progressive in 86.11% of the enrolled subjects. As shown in Figure 1, hearing loss onset was more than 15 years prior to implantation (80.55%) in the majority of cases, and only 11.11% of cases preserved residual hearing in the implanted ear. A total of 27.77% of patients underwent speech therapy after implantation, and 5 CI users (12.91%) developed V1 failure during follow-up, with a mean time of 4.2 years after implant surgery; none of the patients had undergone revision surgery.

**Figure 2:**
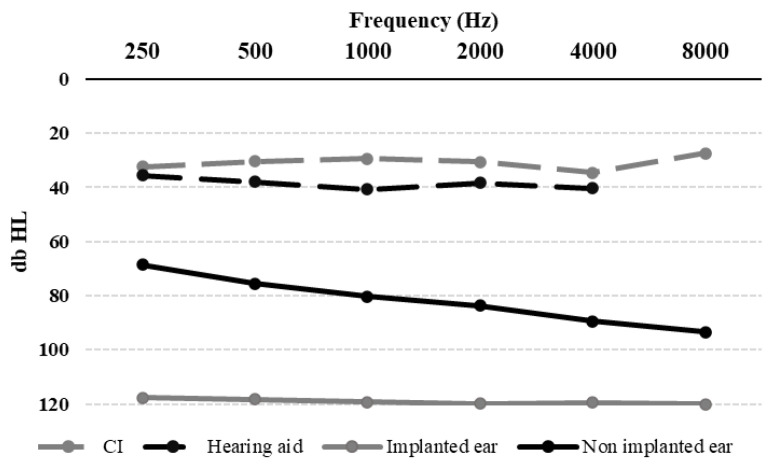
Mean baseline and aided hearing thresholds for both implanted and non-implanted ears.

Statistical analysis of hearing thresholds (Figure 2) revealed that patients with cochlear implants performed significantly differently than those using hearing aids. This distinction was evident in both baseline measurements (*p* = 0.01) and while using the devices (*p* < 0.00001).

**Figure 3:**
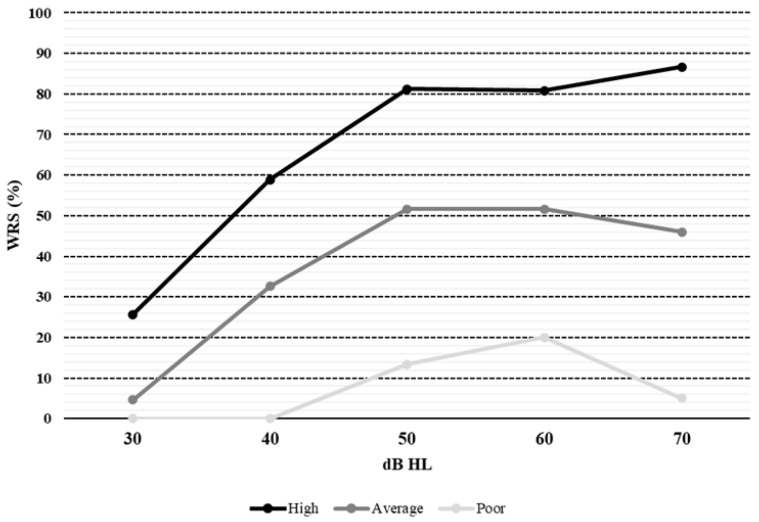
Mean aided speech audiometry curves.

According to speech audiometry results with CI, 16 cases were classified as high, 20 as average, and 3 as poor performers. Mean speech perception outcomes were 69.74% in implanted ears and 70.66% in ears with hearing aids (*p* = 0.9), with 9 out of 16 high performers able to reach 100% speech discrimination and a mean SRT of 40.28 ± 10.42 dB HL; bimodal and unilateral CI users presented a mean maximum speech discrimination of 69.52 ± 24.38 and 61.66 ± 17.49 dB HL, respectively, without a significant difference (*p* = 0.33). No correlation was found between age, duration of deafness before implantation, and maximum speech perception achieved (*p* > 0.05). Conversely, a significant relationship between speech therapy and better speech recognition (*p* = 0.044) was observed. CI wear time was not a predictor of speech perception outcomes (*p* = 0.3594, OR = 1.0794, C.I. = 0.91–1.27). Mean speech audiometry curves (Figure 3), calculated from speech performance with CI at different intensities, showed a rollover phenomenon in poor performers and a plateau effect in average performers. In contrast, the mean curve of high performers showed a steeper morphology, albeit with a shape distinct from that of physiological hearing (S-shaped curve) (*p* < 0.0001). SRT and WRS were observed to predict speech audiogram curves, with a lower SRT (OR = 0.88, C.I. = 0.79–0.98, *p* = 0.006) and higher WRS (OR = 0.88, C.I. = 0.79–0.98, *p* = 0.006) associated with a better shape of the speech curve (OR = 1.06, C.I. = 1.02–1.11, *p* = 0.006).

**Figure 4:**
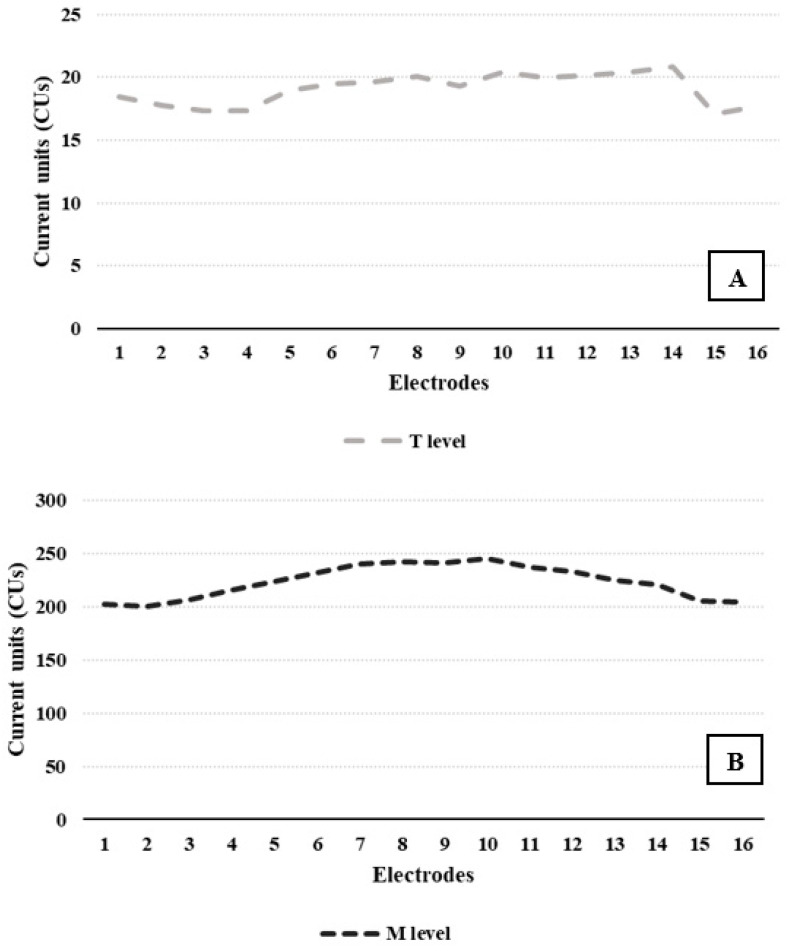
Mean T-level (**A**) and M-level (**B**) for all electrodes of the CIs.

Figure 4 depicts the mean T-levels and M-levels for all electrodes of the total cohort. All maps showed activation of all electrodes, except for four subjects, where it was necessary to deactivate electrodes 15 and 16. This resulted in T-level and M-level values of 19 and 223 CUs, respectively, and a mean dynamic range of 204 CUs. No direct correlation was found between the mean T-levels (ρ = 0.01, *p* = 0.9), M-level (ρ = −0.07, *p* = 0.65), dynamic range (ρ = −0.06, *p* = 0.69), and maximum recognition score. High performers showed a slightly tighter median EDR (164 CUs) than the remaining cohort (*p* > 0.05). No significant difference median WRS was found between patients with a T fixed at 10% of the M level and those with a T at 0 CUs.

We performed further analysis after clustering electrodes into three groups according to their position along the cochlea and presumed frequency band stimulation (1–6 for low, 7–11 for medium, and 12–16 for high frequencies). Mean T-levels, M-levels and the dynamic range of all three groups did not predict either the “degree” of performance or the shape of the speech audiogram curve (*p* > 0.05).

## 4. Discussion

Optimizing speech recognition skills in a CI recipient is the main goal of the CI fitting process; through careful, tailored follow-up, audiologists aim to maximize the benefit of CI surgery [30]. However, despite many efforts, the results of cochlear implantation can show a high variability that cannot always be predicted by pre-implant factors [31,32]. In fact, it is clear that post-surgical variables may influence CI users’ overall speech recognition ability and should be considered when audiologists guide patients through the rehabilitation process [33,34]. Among these, an interesting yet still controversial field of research is the dynamic range of the CI map [35,36,37]. Modifying its parameters may affect the quality and quantity of audible sounds, thereby altering the speech perception outcomes of CI users [38]. It remains clear that during the initial phases immediately after CI activation, a progressive increase in EDR may improve word intelligibility and support adaptation to higher levels of electrical stimulation [20]. Conversely, a different scenario occurs in cases of experienced CI users, where a certain level of speech recognition has already been reached, and further improvement is often not achievable. To complicate matters further, each CI manufacturer has its own specific stimulation strategy, fitting parameters, electrode characteristics, and speech processor technology, making direct comparisons between CI recipients’ results difficult.

Our study retrospectively evaluated a cohort of experienced post-lingually, hearing-impaired adults who were implanted with HiRes devices. No significant correlation was found between the T-level, M-level, EDR, and speech perception outcomes (*p* > 0.05), even after clustering electrodes into three groups based on to their position along the cochlea and the presumed frequency band stimulation (*p* > 0.05). Several studies found no strong relationship between EDR and speech recognition abilities [21,22,23,39,40]. Kim et al., studying users of Nucleus devices with ≥5 years of experience, demonstrated that the EDR was weakly associated with PB word and consonant scores, but not with CAP, K-CID, and vowel scores [21]. Another study, analyzing data from 97 users of Cochlear devices with late-onset hearing impairment, concluded that an EDR range of 40–60 current levels was significantly associated with better speech recognition test results [41]. Other authors adopted a different approach, exploring variation in speech perception after intervention on the map’s parameters. For example, Busby and Arora assessed 19 CI patients using the Nucleus CI system with different levels of EDR expansion and compression; they found no significant effects at 30% expansion and 30% compression and a significant decline in scores for an expansion of 60% and 90% on consonant-nucleus-consonant scores in quiet conditions and SNRs for sentences in noise [36]. Instead, Martins et al. explored the performance of 15 adult CI users with a Cochlear device and over 12 months of listening experience. They observed that lowering C-levels resulted in worse speech perception on both monosyllabic and sentence tests; additionally, T-levels above the behavioral threshold improved sound-field thresholds but did not influence performance in speech recognition tests in quiet or noisy conditions [37]. Unfortunately, given the various methodologies, devices, fittings, performance assessments, and recipients’ characteristics, we cannot draw a parallel between all the aforementioned results and our data.

An interesting insight from our investigation stems from the study of the “dynamic” of speech recognition scores; in other words, we sought to better understand how speech discrimination varies across different speech presentation levels. Literature data usually focuses on single speech outcomes, such as SRT or WRS, without analyzing the morphology or the curve in CI users. According to the degree of performance of the CI, we showed a roll-over phenomenon in poor performers and a plateau effect in average performers, while the mean curve of high performers exhibited a steeper morphology (*p* < 0.0001). Furthermore, SRT and WRS were demonstrated to be predictors of speech audiogram curves, with lower a SRT and higher WRS associated with a better shaped speech curve (*p* = 0.006). We speculate that speech curves may not only provide information about performance at different intensities but also indirectly reflect neural reserve, which is essential for coding the spectral information needed for proper speech understanding. However, it is not possible to determine which part of the acoustic nervous system is impaired; one may hypothesize that a reduced number of spiral ganglion cells is responsible for poor CI performance, but a recent meta-analysis by Cheng and Svirsky failed to find such a correlation [42]. Furthermore, although CIs may restore audibility to near-normal thresholds, the psychoacoustic abilities (e.g., spectral and temporal resolution) that are crucial for understanding speech in noise or for music perception are often found to have deteriorated in CI users [43,44]. Reduced spectral information can lead to difficulties in speech discrimination and analysis of frequency components when hearing complex sounds, especially in noisy environments [45]. Previous studies have also shown impaired temporal processing abilities in CI recipients, assessed using gap and temporal modulation detection, pitch and duration pattern tests [46,47,48].

This study presents the following limitations. First, the retrospective nature of the investigation and the relatively small number of participants did not allow us to consider our results representative of the CI population. Secondly, we focused on investigating the role of EDR, since the *IDR* was maintained at 60 dB in all patients. In fact, an *IDR* of 60 dB is the current default within the latest Advanced Bionics fitting software. In an Advanced Bionics device, an *IDR* range of 65–80 dB can be used to optimally perceive monosyllables in quiet settings; however, any expansion above 65 dB should be evaluated for an audible noise floor and, if acceptable, offered to CI users in clinical settings can provide feedback. In noisy contexts, an *IDR* of 50 dB provided optimal outcomes [22].

Thirdly, we did not perform speech-in-noise tests, which may better reflect speech discrimination outcomes in a more realistic situation. In real-life scenarios, background noise often affects speech intelligibility, and it would be valuable to investigate how CI fitting and other factors influence speech recognition in noisy environments. However, given the lack of evidence for a direct association between EDR and speech in quiet contexts in our cohort, it is unlikely to find a correlation with speech-in-noise discrimination.

Finally, we also consider that our sample is composed only of experienced adult CI users; all of whom showed stable—although not always optimal—results, with the majority attaining a WRS ≥ 70%. In line with other studies [49], post-implantation speech therapy was significantly associated with better speech discrimination (*p* < 0.05), while other prognostic factors were not. This may be interpreted by considering the uniqueness of each CI recipient, whose performance results from a complex interaction among multiple factors that should be considered whenever a clinician refers a patient for cochlear implantation [31,50,51]. In fact, even if negative prognostic factors may guide the audiologist in setting realistic expectations for CI surgery, we underline that unexplained poor outcomes may also arise in “good” candidates for whom no poor outcomes are likely. In light of this, all efforts should be made to provide comprehensive and clear pre-surgery counselling, as well as targeted post-implantation follow-up. The observed rehabilitation adherence rate of 27.77% highlights a significant divergence between theoretical clinical pathways and real-world practice. While certain international healthcare models mandate integrated or residential rehabilitation—often yielding near-universal initial participation—the local landscape is frequently characterized by a structural fragmentation between tertiary referral centers and community-based facilities. This gap is often exacerbated by a shortage of speech-language pathologists specialized in adult auditory rehabilitation. Beyond systemic barriers, this low adherence likely reflects a paradigm shift toward patient-led, autonomous rehabilitation. Post-lingually deafened adults with established linguistic frameworks increasingly favor ecological auditory training and digital health interventions (e.g., mobile applications, audio streaming) over traditional, clinic-based sessions. Furthermore, the phenomenon of rehabilitative burden—the cumulative psychological and logistical exhaustion following a protracted surgical journey—may drive patients to prioritize a rapid return to social normalcy. In many cases, once a functional communicative plateau is achieved, the perceived marginal utility of continued formal therapy diminishes, leading to a strategic discontinuation of clinical follow-ups in favor of informal, real-world auditory integration.

## 5. Conclusions

Our investigation underlines the complexity of CI fitting and its impact on speech perception. While EDR parameters alone did not predict speech recognition outcomes in experienced post-lingually deaf adult CI users, the results underscore that speech therapy and systematic rehabilitation are not merely supplementary but foundational to optimizing performance. Rehabilitation facilitates neural plasticity, enabling the brain to decode the degraded electrical signals from the CI and bridge the gap between audibility and comprehension. This finding is consistent with the broader literature, which highlights that individualized rehabilitation programs are essential for achieving peak outcomes. Speech therapy and rehabilitation efforts proved crucial for optimizing performance.

Furthermore, we propose that clinical assessment should move beyond static single-point measures like SRT or WRS. Instead, a comprehensive analysis of “speech audiogram curves”—which plot the percentage of speech understood across a range of intensities—should be prioritized. The slope of this curve provides a more comprehensive view of global CI performance; a steeper curve indicates a patient’s ability to achieve high intelligibility with minimal increases in volume, reflecting more efficient psychoacoustic processing. Analyzing these curves allows clinicians to visualize how performance shifts as cue audibility varies, offering deeper insights into the patient’s signal elaboration. This data is critical for predicting long-term outcomes and tailoring individualized care plans, such as specific auditory training or map adjustments.

Further studies with larger sample sizes and more homogeneous populations are needed to better understand the interaction between EDR and speech discrimination outcomes.

## Data Availability

The data presented in this study are available upon request from the corresponding author due to the presence of information concerning the personal clinical data of the patients included in the study.

## Abbreviations

The following abbreviations are used in this manuscript:

CI	Cochlear Implant
CU	Clinical unit
*CL*	Current level
SNHL	Sensorineural Hearing Loss
EDR	Electrical Dynamic Range
*IDR*	Input Dynamic Range
PTA	Pure-tone Audiometry
WRS	Word Recognition Score
SRT	Speech Reception Threshold

## References

[1] Boisvert I., Reis M., Au A., Cowan R., Dowell R.C. (2020). Cochlear implantation outcomes in adults: A scoping review. PLoS ONE.

[2] Portelli D., Galletti C., Loteta S., Freni L., Ciodaro F., Alibrandi A., Alberti G. (2025). Patients’ satisfaction and efficacy of modern conventional hearing aids: A comprehensive analysis of the self-reported user experiences in adult people. Braz. J. Otorhinolaryngol..

[3] Berrettini S., Cuda D., Minozzi S., Artioli F., Barbieri U., Borghi C., Cristofari E., Conte G., Cornolti D., di Lisi D. (2025). Cochlear implant procedure. Italian Clinical Practice Guidelines of the Italian Society of Otorhinolaryngology (SIOeChCF) and Italian Society of Audiology and Phoniatrics (SIAF). Part 1: Cochlear implants in adults. Acta Otorhinolaryngol. Ital..

[4] Zeitler D.M., Prentiss S.M., Sydlowski S.A., Dunn C.C. (2024). American Cochlear Implant Alliance Task Force: Recommendations for Determining Cochlear Implant Candidacy in Adults. Laryngoscope.

[5] Kim Y., Han J.H., Yoo H.S., Choi B.Y. (2022). Molecular aetiology of ski-slope hearing loss and audiological course of cochlear implantees. Eur. Arch. Otorhinolaryngol..

[6] Lazard D.S., Vincent C., Venail F., Van de Heyning P., Truy E., Sterkers O., Skarzynski P.H., Skarzynski H., Schauwers K., O’Leary S. (2012). Pre-, Per- and Postoperative Factors Affecting Performance of Postlinguistically Deaf Adults Using Cochlear Implants: A New Conceptual Model over Time. PLoS ONE.

[7] Blamey P., Artieres F., Başkent D., Bergeron F., Beynon A., Burke E., Dillier N., Dowell R., Fraysse B., Gallégo S. (2013). Factors affecting auditory performance of postlingually deaf adults using cochlear implants: An update with 2251 patients. Audiol. Neurootol..

[8] Savvas E., Heslinga K., Sundermann B., Schwindt W., Spiekermann C.O., Koopmann M., Rudack C. (2020). Prognostic factors in cochlear implantation in adults: Determining central process integrity. Am. J. Otolaryngol..

[9] Holden L.K., Finley C.C., Firszt J.B., Holden T.A., Brenner C., Potts L.G., Gotter B.D., Vanderhoof S.S., Mispagel K., Heydebrand G. (2013). Factors affecting open-set word recognition in adults with cochlear implants. Ear Hear..

[10] Plant K., McDermott H., van Hoesel R., Dawson P., Cowan R. (2016). Factors Predicting Postoperative Unilateral and Bilateral Speech Recognition in Adult Cochlear Implant Recipients with Acoustic Hearing. Ear Hear..

[11] Moon I.J., Kim E.Y., Jeong J.O., Chung W.H., Cho Y.S., Hong S.H. (2012). The influence of various factors on the performance of repetition tests in adults with cochlear implants. Eur. Arch. Otorhinolaryngol..

[12] Sladen M., Nichani J., Kluk-de Kort K., Saeed H., Bruce I.A. (2025). Outcomes of attempted hearing preservation after cochlear implantation (HPCI): A prognostic factor (PF) systematic review of the literature. Cochlear Implant. Int..

[13] Zhou X., Seghouane A.K., Shah A., Innes-Brown H., Cross W., Litovsky R., McKay C.M. (2018). Cortical Speech Processing in Postlingually Deaf Adult Cochlear Implant Users, as Revealed by Functional Near-Infrared Spectroscopy. Trends Hear..

[14] Han J.H., Lee H.J., Kang H., Oh S.H., Lee D.S. (2019). Brain Plasticity Can Predict the Cochlear Implant Outcome in Adult-Onset Deafness. Front. Hum. Neurosci..

[15] Stenbäck V., Marsja E., Ellis R., Rönnberg J. (2023). Relationships between behavioural and self-report measures in speech recognition in noise. Int. J. Audiol..

[16] Dorismond C., Patro A., Holder J.T., Perkins E.L. (2023). Correlation Between Quality of Life and Speech Recognition Outcomes Following Cochlear Implantation. Otol. Neurotol..

[17] Berg K.A., Birky H.M., Sevich V.A., Moberly A.C., Tamati T.N. (2025). Sound quality, not speech recognition, explains cochlear implant-related quality of life outcomes. JASA Express Lett..

[18] Migliorini E., van Dijk B., Philips B., Mylanus E., Huinck W. (2024). The relation between cochlear implant programming levels and speech perception performance in post-lingually deafened adults: A data-driven approach. Eur. Arch. Otorhinolaryngol..

[19] Van Opstal A.J., Noordanus E. (2023). Towards personalized and optimized fitting of cochlear implants. Front. Neurosci..

[20] Park B., Thak P.K., Park E., Choi S.J., Lee J., Kwak S., Jung H.H., Im G.J. (2023). Dynamic Range and Neural Response Threshold in Cochlear Implant Mapping Can Be Useful in Predicting Prognosis Related to Postoperative Speech Perception. J. Audiol. Otol..

[21] Kim S.Y., Jeon S.K., Oh S.H., Lee J.H., Suh M.W., Lee S.Y., Lim H.J., Park M.K. (2018). Electrical dynamic range is only weakly associated with auditory performance and speech recognition in long-term users of cochlear implants. Int. J. Pediatr. Otorhinolaryngol..

[22] Nunn T.B., Jiang D., Green T., Boyle P.J., Vickers D.A. (2019). A systematic review of the impact of adjusting input dynamic range (IDR), electrical threshold (T) level and rate of stimulation on speech perception ability in cochlear implant users. Int. J. Audiol..

[23] Fu Q.J., Shannon R.V. (2000). Effects of dynamic range and amplitude mapping on phoneme recognition in Nucleus-22 cochlear implant users. Ear Hear..

[24] Khater A., El Shennaway A., Anany A. (2015). Improvement of cochlear implant performance: Changes in dynamic range. Egypt. J. Otolaryngol..

[25] Holden L.K., Reeder R.M., Firszt J.B., Finley C.C. (2011). Optimizing the perception of soft speech and speech in noise with the Advanced Bionics cochlear implant system. Int. J. Audiol..

[26] Sorrentino F., Gheller F., Lunardi G., Brotto D., Trevisi P., Martini A., Marioni G., Bovo R. (2020). Cochlear implantation in adults with auditory deprivation: What do we know about it?. Am. J. Otolaryngol..

[27] Salvago P., Vaccaro D., Plescia F., Vitale R., Cirrincione L., Evola L., Martines F. (2024). Client Oriented Scale of Improvement in First-Time and Experienced Hearing Aid Users: An Analysis of Five Predetermined Predictability Categories through Audiometric and Speech Testing. J. Clin. Med..

[28] Ovari A., Hühnlein L., Nguyen-Dalinger D., Strüder D.F., Külkens C., Niclaus O., Meyer J.E. (2022). Functional Outcomes and Quality of Life after Cochlear Implantation in Patients with Long-Term Deafness. J. Clin. Med..

[29] Lindquist N.R., Cass N.D., Patro A., Perkins E.L., Gifford R.H., Haynes D.S., Holder J.T. (2022). HiRes Ultra Series Recall: Failure Rates and Revision Speech Recognition Outcomes. Otol. Neurotol..

[30] Hey M., Hocke T. (2025). Optimizing CI Systems for Better Recognition of Soft Speech -the Concept of Broad-Range Mapping. Laryngoscope Investig. Otolaryngol..

[31] Velde H.M., Rademaker M.M., Damen J., Smit A.L., Stegeman I. (2021). Prediction models for clinical outcome after cochlear implantation: A systematic review. J. Clin. Epidemiol..

[32] Shafiro V., Harris M.S., Ramirez B., Du L., Moberly A.C. (2025). Accuracy and variability in clinical predictions of speech recognition outcomes for cochlear implant users. Int. J. Audiol..

[33] Harris M.S., Capretta N.R., Henning S.C., Feeney L., Pitt M.A., Moberly A.C. (2016). Postoperative Rehabilitation Strategies Used by Adults With Cochlear Implants: A Pilot Study. Laryngoscope Investig. Otolaryngol..

[34] Dornhoffer J.R., Reddy P., Ma C., Schvartz-Leyzac K.C., Dubno J.R., McRackan T.R. (2022). Use of Auditory Training and Its Influence on Early Cochlear Implant Outcomes in Adults. Otol. Neurotol..

[35] Loizou P.C., Dorman M., Fitzke J. (2000). The effect of reduced dynamic range on speech understanding: Implications for patients with cochlear implants. Ear Hear..

[36] Busby P.A., Arora K. (2016). Effects of threshold adjustment on speech perception in nucleus cochlear implant recipients. Ear Hear..

[37] Martins K.V.C., Goffi-Gomez M.V.S. (2021). The influence of stimulation levels on auditory thresholds and speech recognition in adult cochlear implant users. Cochlear Implant. Int..

[38] de Quillettes R., Kaandorp M., Merkus P., Kramer S.E., Smits C. (2024). Experienced Adult Cochlear Implant Users Show Improved Speech Recognition When Target Fitting Parameters Are Applied. Ear Hear..

[39] Dawson P.W., Decker J.A., Psarros C.E. (2004). Optimizing dynamic range in children using the nucleus cochlear implant. Ear Hear..

[40] Bento R.F., De Brito Neto R.V., Castilho A.M., Gomez M.V., Sant’Anna S.B., Guedes M.C., Peralta C.G. (2005). Psychoacoustic dynamic range and cochlear implant speech-perception performance in nucleus 22 users. Cochlear Implant. Int..

[41] de Graaff F., Lissenberg-Witte B.I., Kaandorp M.W., Merkus P., Goverts S.T., Kramer S.E., Smits C. (2020). Relationship Between Speech Recognition in Quiet and Noise and Fitting Parameters, Impedances and ECAP Thresholds in Adult Cochlear Implant Users. Ear Hear..

[42] Cheng Y.S., Svirsky M.A. (2021). Meta-Analysis-Correlation between Spiral Ganglion Cell Counts and Speech Perception with a Cochlear Implant. Audiol. Res..

[43] Glasberg B.R., Moore B.C.J. (1989). Psychoacoustic abilities of subjects with unilateral and bilateral cochlear hearing impairments and their relationship to the ability to understand speech. Scand. Audiol. Suppl..

[44] Strelcyk O., Dau T. (2009). Relations between frequency selectivity, temporal fine-structure processing, and speech reception in impaired hearing. J. Acoust. Soc. Am..

[45] Won J.H., Drennan W.R., Rubinstein J.T. (2007). Spectral-ripple resolution correlates with speech reception in noise in cochlear implant users. J. Assoc. Res. Otolaryngol..

[46] Duarte M., Gresele A.D., Pinheiro M.M. (2016). Temporal processing in postlingual adult users of cochlear implant. Braz. J. Otorhinolaryngol..

[47] Shafiro V., Gygi B., Cheng M.Y., Vachhani J., Mulvey M. (2011). Perception of environmental sounds by experienced cochlear implant patients. Ear Hear..

[48] Fraser M., McKay C.M. (2012). Temporal modulation transfer functions in cochlear implantees using a method that limits overall loudness cues. Hear. Res..

[49] Dornhoffer J.R., Chidarala S., Patel T., Khandalavala K.R., Nguyen S.A., Schvartz-Leyzac K.C., Dubno J.R., Carlson M.L., Moberly A.C., McRackan T.R. (2024). Systematic Review of Auditory Training Outcomes in Adult Cochlear Implant Recipients and Meta-Analysis of Outcomes. J. Clin. Med..

[50] Portelli D., Loteta S., D’Angelo M., Galletti C., Freni L., Bruno R., Ciodaro F., Alibrandi A., Alberti G. (2025). ChatGPT and Microsoft Copilot for Cochlear Implant Side Selection: A Preliminary Study. Audiol. Res..

[51] Portelli D., Lombardo C., Loteta S., Galletti C., Azielli C., Ciodaro F., Mento C., Aguennouz M., Rosa G.D., Alibrandi A. (2024). Exploring the Hearing Improvement and Parental Stress in Children with Hearing Loss Using Hearing Aids or Cochlear Implants. J. Clin. Med..

